# A Survey on M2M Systems for mHealth: A Wireless Communications Perspective

**DOI:** 10.3390/s141018009

**Published:** 2014-09-26

**Authors:** Elli Kartsakli, Aris S. Lalos, Angelos Antonopoulos, Stefano Tennina, Marco Di Renzo, Luis Alonso, Christos Verikoukis

**Affiliations:** 1 Department of Signal Theory and Communications (TSC), Technical University of Catalonia (UPC-BarcelonaTECH), C./ Esteve Terradas 7, Castelldefels 08860, Barcelona, Spain; E-Mails: aristeidis.lalos@tsc.upc.edu (A.S.L.); luisg@tsc.upc.edu (L.A.); 2 Telecommunications Technological Centre of Catalonia (CTTC), Av. Gauss 7, Castelldefels 08860, Barcelona, Spain; E-Mails: aantonopoulos@cttc.es (A.A.); cveri@cttc.es (C.V); 3 WEST Aquila slr, Via Giovanni Gronchi 18, L'Aquila 67100, Italy; E-Mail: tennina@westaquila.com; 4 Laboratory of Signals and Systems (L2S), CNRS - SUPELEC - Univ. Paris-Sud XI, 3 Rue Joliot-Curie, 91192 Gif sur Yvette, France; E-Mail: marco.direnzo@lss.supelec.fr

**Keywords:** Machine-to-machine (M2M), mHealth, eHealth, healthcare, WBAN, end-to-end communications, IEEE 802.15.6

## Abstract

In the new era of connectivity, marked by the explosive number of wireless electronic devices and the need for smart and pervasive applications, Machine-to-Machine (M2M) communications are an emerging technology that enables the seamless device interconnection without the need of human interaction. The use of M2M technology can bring to life a wide range of mHealth applications, with considerable benefits for both patients and healthcare providers. Many technological challenges have to be met, however, to ensure the widespread adoption of mHealth solutions in the future. In this context, we aim to provide a comprehensive survey on M2M systems for mHealth applications from a wireless communication perspective. An end-to-end holistic approach is adopted, focusing on different communication aspects of the M2M architecture. Hence, we first provide a systematic review of Wireless Body Area Networks (WBANs), which constitute the enabling technology at the patient's side, and then discuss end-to-end solutions that involve the design and implementation of practical mHealth applications. We close the survey by identifying challenges and open research issues, thus paving the way for future research opportunities.

## 1. Introduction

Machine-to-Machine (M2M) communications is an emerging technology that envisions the interconnection of machines without the need of human intervention. The main concept lies in seamlessly connecting an autonomous and self-organizing network of M2M-capable devices to a remote client, through heterogeneous wired or wireless communication networks. An intelligent software application is usually employed at the remote client to process the collected data and provide the end user with a set of smart services and a practical interface. Although the idea of telematics and telemetry applications is not new, the widespread use of Internet, along with the trend for ubiquitous connectivity, especially via wireless communication systems, have placed M2M systems on the spotlight of attention for both academia and industry.

The increasing interest on M2M communications poses significant challenges that need to be met. A key issue to be handled is the large number of devices that must be supported in an M2M network, since market predictions estimate that the number of M2M-enabled devices with Internet connectivity will reach up to 50 billion by the end of 2020 [1]. Regardless of the exact figures, the growth rate is impressive, and major efforts are required to provide scalable solutions that support the increasing number of devices with diverse characteristics and requirements. Another challenge stems from the multitude of technical solutions that can be employed in M2M systems. Depending on the application deployment, different approaches may be adopted for the interconnection of M2M devices, such as wired or wireless technologies, short-range or long-range communications, and solutions based on existing open communication standards or proprietary technologies.

The above challenges stress the imperative need for standardization of M2M communications [2]. To this direction, the European Telecommunications Standards Institution (ETSI) has established the M2M Technical Committee which aims to provide an end-to-end view of M2M standardization, focusing on the interoperability of M2M devices with existing standards. In July of 2012, ETSI and six other major standards development organizations (ARIB and TTC of Japan, ATIS and the TIA of the USA, CCSA of China, and TTA of Korea) joined their efforts in the one M2M initiative, under the goal of creating a single universal standard for M2M communications [3]. This global standardization effort is crucial to enable the integration of heterogeneous technologies in order to achieve seamless end-to-end connectivity, removing potential barriers to market growth.

The penetration of M2M solutions for monitoring and remote control in a wide range of markets, including industrial automation, security and surveillance, smart metering, energy management, and transportation, generates great business opportunities. The application of M2M enabling technologies to the healthcare sector, in particular, is expected to be one of the major M2M market drivers: market projections forecast that more than 774 million health-related devices with M2M connectivity will be available by 2020, yielding a total revenue of 69 billion euros in that year [4].

The use of mobile and wireless communication technologies to facilitate and improve healthcare and medical services, referred to by the term mHealth, is bringing a shift to healthcare delivery. The mHealth field can be considered as a subset of telemedicine, which is a broad term referring to the delivery of healthcare services between at least two remote locations, involving the communication between healthcare providers or the interaction between a healthcare provider and a patient [5]. Telemedicine can transform healthcare delivery across several medical disciplines, from cardiology and surgery to ophthalmology and psychiatry. The potential mHealth application scenarios are numerous, including the active management of diseases such as diabetes (e.g., by measuring blood sugar levels and controlling the insulin dosage accordingly), the support for independent aging to the elderly (e.g., by tracking their medication intake and their activity level) and the monitoring of personal fitness activities to improve health and well-being (e.g., by logging health and fitness indicators during workouts) [6]. As the sensing technology advances, future mHealth applications are expected to emerge [7,8], such as cancer detection (through the monitoring of cancer cells), management of heart condition (through the monitoring of biochemical markers) and vision enhancement (through implanted retina prosthesis chips), to name a few. Overall, mHealth can offer significant benefits for both patients and healthcare providers, ensuring enhanced quality, efficiency, flexibility and cost reductions in healthcare delivery.

The M2M paradigm in the context of mHealth involves the use of appropriate sensor devices on patients. The medical sensors, placed in the vicinity of, or inside, the human body, are usually interconnected through a short-range wireless technology, thus forming a Wireless Body Area Network (WBAN). An M2M-enabled gateway node collects all the sensory data from the WBAN and forwards them to a remote online server, where processing and integration with medical-related software applications take place. The connection of the gateway to the Internet is generally based on long-range communication access technologies for Wireless Local/Metropolitan Area Networks (WLANs/WMANs). The M2M technology paves the way for new possibilities for mHealth applications, by enabling the remote monitoring of vital signals [9], the early detection of critical conditions and the remote control of certain medical treatments [6]. The collection of large amount of data and their timely delivery to the healthcare providers in an unobtrusive way for the patient and without the need for human interaction can significantly facilitate the management of chronic diseases and speed up the diagnosis of critical conditions.

In the recent years, the research community has been motivated by the diversity of applications, the promising benefits and the potential market opportunities of mHealth M2M solutions. The main technological challenges for M2M communications, the most representative usage models and the status of global standardization efforts are discussed in [10]. Focusing on the emerging M2M technologies for mHealth applications, a technical discussion on the communication network design is given in [11], but generally most of the related work adopts a high-level approach. In [12], some interesting challenges from the network perspective are identified, while interoperability issues and recent standardization efforts are presented in [13]. On a different level, a lot of research activity has been focused on the body area domain, on the design of medical sensor devices [14] and on the main advances and challenges in the field of WBANs [15–18].

However, recent important work in the rapidly growing field of WBAN communication technologies have not been included in the aforementioned works, while the end-to-end holistic M2M approach is barely considered. In this context, this paper aims to provide a comprehensive survey on M2M systems for mHealth applications from a wireless communication perspective. This paper discusses many aspects of the M2M ecosystem in the healthcare domain, however, the main novel contribution is threefold:

- a systematic review of existing physical (PHY) layer technologies for WBANs, which, to our knowledge is not available in the literature,
- an up-to-date survey of the key Medium Access Control (MAC) layer design approaches to tackle the specific challenges of mHealth applications in WBANs, and
- an end-to-end perspective of M2M systems for mHealth, focusing on the integration and convergence of different communication technologies employed in WBANs and long-range communication networks and the presentation of testbed implementations.

The remainder of this paper is organized as follows. First, the high-level ETSI architecture for M2M systems is described in Section 2, whereas the main design challenges for end-to-end mHealth applications are discussed in Section 3. Section 4 is dedicated to the most recent developments in wireless technologies for WBANs. After a brief description of the IEEE 802.15.6 standard for WBANs, the focus is laid on channel modeling, PHY technologies and MAC strategies. Then, Section 5 is devoted to end-to-end connectivity, focusing on integration challenges, existing testbeds and current research projects. Finally, some concluding remarks are given in Section 6.

## 2. M2M Network Architecture

In the recent years, ETSI has been actively engaged in the development of a standard for M2M systems, with the objective to ensure interoperability between the diverse M2M components and the already existing technologies. To this direction, ETSI proposes a high-level horizontal architecture, dividing the system into three domains: (i) the device and gateway domain where the M2M devices communicate with a gateway through short-range area networks; (ii) the network domain that connects the gateway to the applications through long-range access and core communication networks; and (iii) the application domain where various application services are defined depending on the use case [19].

**Figure 1.**
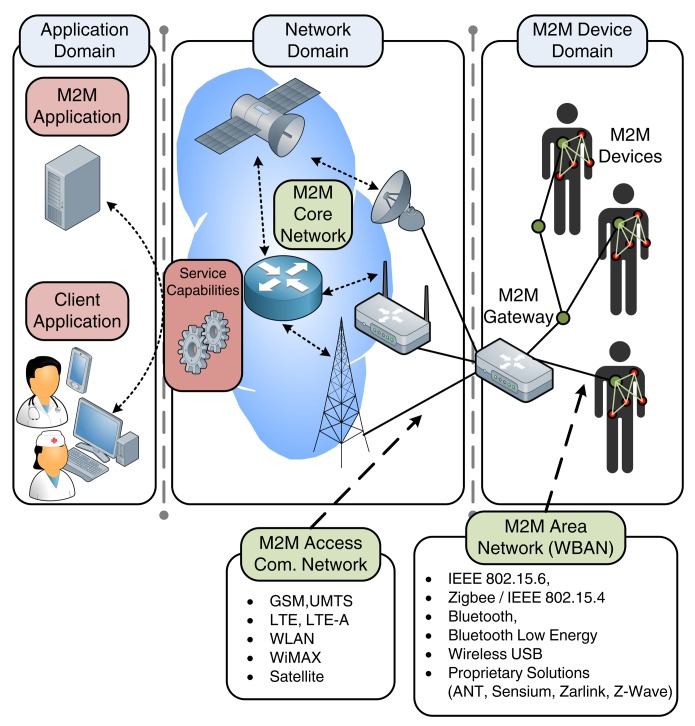
Simplified Machine-to-Machine (M2M) architecture for wireless connectivity in mHealth scenarios.

Figure 1 illustrates the architecture of a wireless M2M system for mHealth applications. The ETSI framework consists of five key elements as described below [19]:

- The M2M devices, which are devices capable of transmitting data autonomously or after receiving a data request. In the context of mHealth applications, the M2M devices are principally low-power medical sensors and actuators, placed close to or inside the patient's body (wearable or implanted sensors, respectively). Each device has an embedded wireless communication module, enabling their interconnection into a short-range network, as described in continuation.
- The M2M Area Network, also known as capillary network, which is the short-range network interconnecting the M2M devices and providing a link to the M2M gateway. Focusing on mHealth scenarios where wireless connectivity is considered among the body sensors, WBANs are the enabling technology for the M2M area network. Hence, in the rest of this paper, the terms M2M area network and WBAN will be employed interchangeably. IEEE 802.15.6 [20] is an emerging communication standard developed specifically for WBANs and suitable for healthcare applications. Other candidate technologies for the capillary network include ZigBee and IEEE 802.15.4, Bluetooth, and Bluetooth Low Energy. Proprietary solutions are also available, such as ANT, Sensium, Zarlink and Z-Wave.
- The M2M Gateway, which acts as a proxy between the M2M devices (interconnected through the WBAN) and the network domain. Practically, the gateway must be a portable device with advanced processing capabilities and multiple radio interfaces, able to operate in technologies employed by both the WBAN and the communication network. Typical example of M2M gateways include smartphones, Personal Digital Assistants (PDAs) and smart watches.
- The M2M Access Communication Network, which connects the M2M gateway to the Internet, eventually reaching the M2M application server. The communication network can be broken down to the access network and the core network. Common wireless technologies employed by the access network include Long Term Evolution (LTE), WiMAX and IEEE 802.11 WLAN. Core network technologies provide IP connectivity and include 3GPP, 3GPP2 and ETSI TISPAN core network systems.
- The M2M Application Server, which is the middleware layer that provides data to the specific business applications.

## 3. Design Challenges for mHealth Applications

The previously described M2M system architecture can be employed in the context of wireless mHealth applications, aiming to form a communication bridge between the patients and the healthcare providers. At the patient's side, we consider WBANs, defined as low-power short-range networks deployed in the vicinity of the human body, as the prevalent technology for the M2M area (or capillary) network. The nature of WBANs poses inherent limitations not present in regular sensor networks, due to the specific characteristics and requirements of the employed M2M sensor devices. Furthermore, to provide end-to-end connectivity, the integration of different medium and long range technologies must be ensured, guaranteeing reliable and secure communication. The key design characteristics and challenges that must be taken into account at different stages of the M2M system, in order to design efficient techniques and communication protocols are summarized next [15,16,21,22]:

- *Heterogeneous devices and traffic*: In mHealth applications, the M2M devices are usually medical sensors that can be either attached on (wearable or on-body), or placed inside (implantable or in-body) the human body. These devices are capable of establishing wireless communication links, in order to enable continuous patient monitoring and provide real-time feedback to the responsible healthcare provider. Based on their operation, they can be classified as sensors or actuators. Sensors are used to measure external (e.g., motion, location, environmental temperature, *etc.*) or internal (e.g., heart beat, body temperature, muscle movement, brain activity, *etc.*) parameters of the human body. Actuators perform specific actions according to data received from the sensors or inserted by the end user (e.g., a pump for the administration of insulin based on blood glucose measurements). Unlike typical sensor networks, the number of devices in a WBAN is relatively small (*i.e.*, usually restricted within the range of 20–50), with each device having a unique function. Table 1 contains a list of commonly employed sensors, emphasizing their diverse communication characteristics and requirements. As observed from the table, different types of data must be supported, ranging from plain messages to real-time audio and video content and continuous waveform signals, such as Electrocardiogram (ECG), Electromyograph (EMG), *etc*. Consequently, WBANs must be able to handle heterogeneous traffic and support a diverse range of transmission rates. Scalability is also a key feature, enabling the seamless addition or removal of nodes without affecting the network's operation.
- *Wireless propagation characteristics*: The propagation characteristics for most well established wireless technologies (e.g., cellular, WLANs, *etc.*) have been extensively studied. In the context of sensor networks, several studies have been made with respect to the signal propagation in different communication media [24]. Nevertheless, wireless communication close or inside the human body introduces new challenges due to the different propagation characteristics of the body area environment. Even though the distances between the nodes are small, WBAN links suffer from high propagation losses, mainly due to user activity, in the case on on-body communications, and power absorption by the human tissue, in the case of implants. In addition, patient mobility and frequent changes in body posture can affect the quality of the wireless link.
- *Energy efficiency*: Even though energy efficiency is a desirable feature in all parts of an M2M communication system, it becomes of paramount importance in WBAN environments. To enhance comfort and unobtrusive wearability, body sensor devices must have a very small form factor, which unavoidably has a direct impact on the size and the capacity of the available energy sources (*i.e.*, the batteries). Furthermore, there is an imperative need for prolonged battery lifetime, sometimes in the order of years, since battery replacement in the context of healthcare applications is not a trivial issue, especially in the case of implantable devices. Hence, efficient transceiver design, to ensure low-power consumption, and energy-aware communication protocols are essential.
- *Quality of Service (QoS)*: The QoS requirements of an M2M mHealth system strongly depend on the application, given the very heterogeneous traffic characteristics that range from low-rate, predictable traffic to bandwidth-hungry real-time applications. Nevertheless, low end-to-end delay can be considered the most stringent QoS requirement in mHealth scenarios, especially when real-time monitoring is involved. As reported in [8], latency in medical applications should not exceed 125 ms, whereas bit error rates in the range of 10^−3^ to 10^−10^ should be supported, depending on the application. In addition, a critical issue in patient monitoring is the handling of alarm messages, generated by a sensor device when, for example, a sampled value exceeds some predefined limits. These unpredicted uplink data must be guaranteed timely delivery, since they can be closely associated to urgent, life threatening conditions for the patient. Typically, applications are classified into different service classes, based on their particular QoS requirements. Then, advanced scheduling and routing schemes are employed to provide different levels of priority to each service class.
- *Reliability*: Closely connected to QoS guarantees, reliability is another important issue in mHealth applications. We define reliability as the guaranteed end-to-end delivery of the transmitted data, from the patient to the medical personnel, or vice versa. The most vulnerable part of the M2M architecture in terms of reliability is the patient-side WBAN, due to the error-prone WBAN channels, which must take into account the particular propagation characteristics of implanted and on-body wireless links and patient mobility.
- *Interference and coexistence*: WBANs operate on very low transmission power, given the short-range connectivity requirements and the strict regulations on the acceptable Specific Absorption Rate (SAR) limits (to reduce the risks from radiation exposure). Hence, on one hand, interference caused by WBANs is relatively low. On the other hand, interference tolerance and coexistence with other wireless systems can be a challenging issue, especially for WBANs operating at the industrial, scientific, and medical (ISM) frequency band and deployed in environments with many interfering sources.
- *Topology*: Typically, the most common WBAN configuration is the star topology, where all the sensor devices are connected to the WBAN coordinator via a direct link. However, it is also possible to use part of the deployed sensors as relays [25], to improve connectivity in case of poor quality direct links, thus enhancing reliability [26,27] and energy efficiency [28]. In the M2M access communication network, a point-to-point medium/long-range connection between the WBAN gateway and the core network is usually assumed (e.g., through a WLAN access point or an LTE base station), without excluding the possibility of more complex topologies. For instance, it is possible to exploit the presence of multiple WBANs [29] or ambient sensor network deployments [30] in order to establish multihop links, enabling the design of efficient routing and cooperative schemes.
- *Security*: A key challenge of mHealth applications is to guarantee of the confidentiality of medical information, which faces additional threats due to the M2M architecture, mainly due to: (i) the wireless transmission of medical-sensitive data; and (ii) the data storage in multiple locations, both locally and in remote servers and devices. Hence, a need for a holistic approach is generated in order to ensure secure M2M communication [31]. To this direction, new schemes have to be designed, taking into account the specific characteristics of different technologies, in order to create interoperable and technology-independent protocols and security mechanisms.
- *Technology integration*: The wireless technologies employed in the different stages of the M2M system architecture have diverse characteristics and challenges that are often studied separately. Nevertheless, the integration of these technologies into a unified mHealth application is an open issue that must be considered carefully. Therefore, in order to guarantee end-to-end QoS, scalability and ubiquitous connectivity, it is important to adapt the access communication technologies, such as LTE, WiMAX and IEEE 802.11 WLAN, to the characteristics of WBANs, taking into account the requirements of pervasive mHealth applications.

## 4. WBANs: The Key M2M Area Network Technology for mHealth Applications

As seen in the previous section, many of the challenges present in M2M mHealth systems are related to the area network domain, thus stressing the important role of WBAN technologies. Hence, in this section, we aim to provide an overview of the research work on some key aspects of WBAN design. First, we provide a concise description of IEEE 802.15.6, which is the latest IEEE standardization activity targeted specifically to WBANS. Then, we describe the main channel modeling methods and propagation characteristics for body area communications. In continuation, we focus on different PHY layer technologies that can be employed in WBANs. Finally, this section ends with an up-to-date survey of the most representative MAC mechanisms designed in the context of mHealth applications in WBANs.

### 4.1. IEEE 802.15.6 Standard for WBANs

In an effort to tackle the specific requirements of WBANs, the IEEE 802.15.6 standard has recently been issued for short-range wireless communications in the vicinity of, or inside, the human body. A key characteristic of IEEE 802.15.6 is that it supports operation at very low transmission powers, as well as macroscopic and microscopic power management through hibernation and sleep modes, respectively, in an effort to increase battery lifetime and comply with the safety regulations limits on the maximum acceptable absorption level (*i.e.*, SAR) for in-body communications. It also provides QoS guarantees for the prompt delivery of alarms in emergency situations and employs robust security mechanisms to provide privacy and confidentiality protection of the medical data.

The standard provides specifications for the PHY and the MAC layers. With respect to the PHY layer, three different technologies are supported: (i) the Narrowband (NB) PHY, which introduces low control overhead, very low peak power consumption and robustness against interference; (ii) the Ultra-Wideband (UWB) PHY, based on a technology for transmitting information over a large bandwidth, offering high performance, robustness, low complexity and ultra-low power operation; and (iii) the Human Body Communications (HBC) PHY, which uses the human body as a means of propagation for the data transmission, through the galvanic coupling of signal currents.

Operation at multiple frequency band is supported, starting from the Medical Implant Communications Service (MICS) band of 402–405 MHz, reserved for medical implant communication, up to the unlicensed 2.4 GHz ISM band. Table 2 summarizes the frequency bands supported by each PHY layer technology, as well as the range of achievable transmission rates.

With regard to the MAC layer, IEEE 802.15.6 defines eight levels of user priorities, with level 0 corresponding to the lowest priority class and level 7 being assigned to the highest priority traffic for emergency situations or medical implant event reports. The WBAN operates in an extended star topology, with all nodes connected directly, or through a single relay node, to the coordinating node, denoted as the hub. The standard considers both contention-based and contention-free channel access.

Two random access schemes for contention-based access are supported: (i) Carrier Sense Multiple Access with Collision Avoidance (CSMA/CA); and (ii) slotted Aloha. In CSMA/CA, a node has to sense the medium idle before transmitting and executes a backoff mechanism to reduce the collision probability and resolve contentions in case of simultaneous transmission attempts. In slotted Aloha, time is divided into slots and nodes attempt transmission at the beginning of each slot with a given probability that depends on the user priority.

The standard also supports three contention-free access modes that require centralized control from the WBAN hub: (i) improvised access, which is a polling scheme for immediate of future allocation for both uplink and downlink; (ii) scheduled access, which are periodic allocations for uplink, downlink or bidirectional transmissions that are negotiated between the nodes and the hub during the association phase; and (iii) unscheduled access, which is a best-effort version of the scheduled access.

As far as security is concerned, the standard defines three different connectivity levels: (i) unsecured communication; (ii) authentication only; and (iii) authentication and encryption.

### 4.2. WBAN Channel Modeling

Accurate and reliable channel models are crucial for the design and evaluation of transmission and reception techniques employed at the PHY layer. Hence, considerable efforts have been made to define the characteristics of the body area propagation environment through extensive measurements and simulations. In this section, we present the most important results related to the channel modeling for on-body, in-body and HBC communications in WBANs, along with the different factors that affect the propagation characteristics.

#### 4.2.1. On-Body Radio Propagation Model

This section summarizes the most important findings related to the path loss and the small-scale fading of on-body radio channels.

##### Path loss Model

According to [32–35], propagated waves are more likely to diffract around the human body, rather than penetrate it. Hence, traditional formulas for wireless communications can be applied, such as the Friis equation that caclulates the path loss as a function of the distance *d* [36]:

(1) $$\text{Path loss}∼G_{r}G_{t}{\left(\frac{\lambda }{4\pi }\right)}^{2}{\left(\frac{1}{d}\right)}^{n}$$

where *G_t_* and *G_r_* are the respective transmitter and receiver antenna gains, λ is the wavelength, and *n* is the path loss exponent that depends on the propagation environment.

In free space propagation environments, the path loss exponent is equal to *n* = 2. In the context of WBANs, however, *n* has a higher value, depending on the scenario. In particular, when the transmit and receive antennas are placed at the same side of the body (Line-of-Sight-LOS situation), it has been estimated that the values of *n* range between 3 and 4, depending on the position of the device (*i.e.*, the path loss on the arm is lower that the one on the trunk). Then, in Non-LOS (NLOS) cases that occur when the antennas are placed at different sides of the body, *n* assumes higher values, between 5 and 7.4 [33,35].

It is important to mention that in the context of WBANs, frequency does not significantly affect the path loss [37]. On the other hand, the current user activity and the antenna height (defined as the distance from the body) are the two major contributors to the path loss characteristic of wearable WBANs. Regarding the former, the impact of user activity (*i.e.*, walking) on the path loss has been experimentally studied in [38]. The obtained results show that the subject's mobility causes significant deviations in the path loss, with respect to the average path loss values obtained when the subject remains static. Concerning the antenna height, the authors in [39] claim that the closer the antenna is to the body, the higher is the path loss. A difference of more than 20 dB can be obtained by comparing the path loss for antennas placed at 5 mm and 5 cm from the body surface.

##### Small-scale fading

Small-scale fading is different in on-body WBANs with respect to other wireless environments. Several distributions have been proposed to model small-scale fading in different frequency bands [15,40]. However, traditional Rayleigh and Rician distributions have been proven inefficient, while the lognormal distribution seems to be the best fit [41]. In addition, measurements in [40] have indicated that the multipath components can be divided into two clusters. The first cluster is due to the initial wave diffraction around the body and the second is due to reflections on the ground. The correlation between the bins of the two clusters is very weak, and therefore the bins from the different clusters can be considered as statistically independent. On the other hand, the different delay bins of the same cluster are highly correlated. This can be explained by considering that when the path length is very short, the multipath components have overlapping path trajectories. In a real situation, however, the reflections from the scatterers in the surrounding environment [35] should be also taken into account.

In conclusion, the recommended body area channel model for wearable WBANs should consider a fixed two cluster mutlipath model containing: (i) a dominant direct component; (ii) a set of early reflected waves adding (possibly distructively) to the dominant wave; and (iii) a set of excessively delayed waves, adding incoherently to the dominant wave. The dominant wave amplitude is determined by an exponential path loss and a log normal fading coefficient that is strongly correlated with the fading coefficients of the early delayed waves.

#### 4.2.2. In-Body Radio Propagation Model

As mentioned in the previous section, communication of medical implantable devices is restricted to the MICS frequency band of 402–405 MHz. In this frequency range, the human body can be considered as an inhomogeneous medium consisting of multiple tissue layers with different electrical characteristics. Therefore, it behaves as a communication channel where losses are mainly due to the power absorption by the different tissue layers.

Radio propagation through human tissue has been extensively studied in the literature ([42–46]) and depends on many parameters, including, among others, sex, age, location of the sensor in the human body and general posture of the body. The derivation of an exact expression for the in-body propagation model is extremely difficult. Instead, the SAR level is employed as a standard measure of how much power is absorbed by the tissue. Compared to the free space propagation, an additional 30–35 dB path loss is noticed at small distances. Measurements in [44–46] demonstrated that direct communication between implanted nodes suffers from a lower path loss with respect to the link establishment between an implant and a node in body surface. Finally, it has been shown that the shape and the position of the patient's body has a significant impact on the radiation pattern of an implanted radio transmitter [47].

#### 4.2.3. HBC Propagation Model

Apart from the propagation of radio waves in human body, WBANs support HBC, based on data transmission by capacitive and galvanic coupling, as shown in [48–51]. The HBC propagation characteristics are mainly determined through measurements aiming to study the electrical behavior of the human body. In [48], it has been shown that the transmitted signal level experiences high variations at different locations of the body. For example, short-range links between devices placed on the thorax or on the limbs have shown excellent transmission characteristics, whereas high attenuations that prohibit long-distance transmissions have been measured for extremities and joints.

### 4.3. PHY Technologies for WBANs

PHY layer technologies determine the way in which the source data is converted into electrical signal for transmission. In order to increase the energy and bandwidth efficiency, as well as the transmission reliability, a series of signal processing functions must be carried out, including channel coding and decoding, modulation and demodulation, filtering and frequency conversion, synchronization, channel estimation and equalization. All these operations are executed at both the transmitter and the receiver of a digital communication system, which are typically implemented together in a single device, referred to as the transceiver. This section aims to provide a survey of the main challenges in the optimization of different transceiver parts in WBANs for mHealth applications, filling the respective gap that, to our knowledge, exists in the literature.

In accordance to the PHY layer technologies supported by the IEEE 802.15.6 specification, as mentioned in Section 4.1, we divide this section into three parts, devoted to NB, UWB and HBC transceivers, respectively. Particular emphasis will be placed on UWB transceivers, since it is a promising technology for WBAN applications, due to its high energy efficiency and low complexity.

#### 4.3.1. NB Technologies

NB transceiver technologies have been traditionally employed in short and medium range technologies (e.g., Zigbee, Bluetooth, IEEE 802.11 WLANs) and are widely used in commercially available products. In WBANs, NB systems can operate at different frequency bands (Table 2) and are suitable for both on-body and in-body communications. Most existing works on NB transceiver design are focused on the implementation of energy-efficient modulation and demodulation schemes, and their contributions are summarized in the rest of this section.

Two main classes of NB receivers for on-body WBANs may be found in the literature: (i) ZigBee receivers, equipped with quadrature mixers that support Quadrature Phase Shift Keying (QPSK) modulation. The power expensive Phase-Locked Loops (PLLs) and the quadrature oscillators decrease the energy efficiency of NB receivers with respect to UWB or HBC receivers; (ii) Receivers that use regenerative positive feedback to achieve gain, and non-coherent detection to improve energy efficiency. The modulation schemes applied in this case are simple amplitude-based signaling schemes such as On-Off-Keying (OOK). The main drawback of these receivers is the poor sensitivity to RF signals, that can cause oscillation. When oscillating, not only is it impossible to receive any signal, but those oscillations are driven against the antenna and retransmitted into the air, causing interference to nearby receivers.

In the case of in-body communications, given that low-power consumption is one of the key requirements, energy-efficient modulations with small envelope variations are favorable. However, when high data rates must be employed, it is of great importance to choose a high-sensitivity modulation and demodulation technique in order to overcome the strong attenuation caused by the human body. The modulation schemes that have prevailed in NB systems for mHealth applications are *π*/4-shift QPSK, offset QPSK, FSK and MSK (Minimum Shift Keying). Oh *et al.* [52] propose a new type of PSK modulation for high data rates, called Phase Silence Shift Keying (PSSK), which is more bandwidth-efficient than orthogonal modulation schemes such as OOK. Under an Additive White Gaussian Noise (AWGN) channel model with path loss for the human body, the authors in [52] show that the BER of an 8-PSSK is lower than that of various sinusoidal carrier-based modulation schemes. The authors in [53] propose a simple ultra-low power hardware design for an FSK/MSK direct modulation transmitter in medical implant communications. Finally, an extended protocol that uses a rateless code with FSK modulation in order to overcome the reliability and power cost concerns in implanted sensors has been proposed in [54].

#### 4.3.2. UWB Technologies

UWB transceiver technologies, mainly used for on-body communications, are characterized by very low radiated power (*i.e.*, −41.3 dBm/MHz), low implementation complexity, and harmonic coexistence with other existing wireless technologies. Hence, there is an increasing interest in the use of UWB transceivers in biomedical scenarios.

Based on the method employed to form the carrier signal, the UWB technologies are classified into three groups: (i) Impulse Radio UWB (IR-UWB); (ii) Frequency Modulated UWB (FM-UWB); and (iii) Multi-Band Orthogonal Frequency Division Multiplexing UWB (MB-OFDM UWB). A brief description and related work on each technology are presented in continuation.

##### IR-UWB

**Figure 2.**
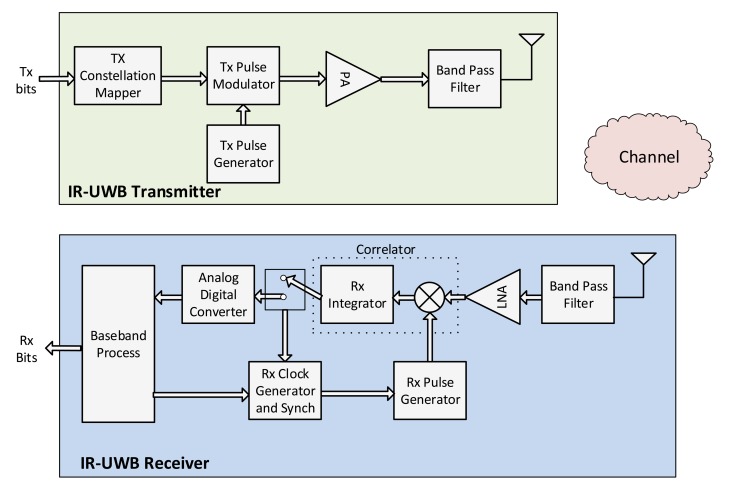
Impulse Radio Ultra-Wideband (IR-UWB) Transceiver.

IR-UWB employs an ultra-short time pulse (in the order of ns) and, therefore, occupies a wide band in the frequency domain. IR-UWB signals are characterized by: (i) very wide bandwidth that can exceed 1 GHz; (ii) very low power spectral density; and (iii) a relatively simple and inexpensive architecture for both transmitter and receiver. A simple transceiver structure for IR-UWB signals is shown in Figure 2.

IR-UWB can support many of the well known modulation schemes that are usually employed in NB systems [55], resulting to a wide range of achieved data rates. In order to demodulate IR-UWB signals, coherent, differentially coherent, and non-coherent detection schemes can be applied. A performance study of an IR-UWB system for WBANs is presented in [56], where the impact of two different modulation techniques on the Bit Error Rate (BER) of different on-body radio links is analyzed. The authors in [57] have studied different (coherent and non-coherent) UWB receivers, implemented according to the IEEE 802.15.4a requirements in different hospital environments, showing that both receivers yield equivalent performance in short-distance LOS links.

The authors in [58] have investigated the problem of pulse distortion due to the interaction between UWB electromagnetic pulses and the human body. By studying the performance of various detection algorithms in the presence of pulse distortion and receiver noise, it has been shown that non-coherent receivers, such as energy detectors and Transmitted-Reference (TR) receivers (In TR systems, two signals—a reference and a data signal—are transmitted per bit. The information is contained in the sign difference of the transmitted signals.), are suitable for WBANs, due to the fact that they do not require any explicit channel estimation (thus consuming less power and reducing complexity). In [59], two non-coherent modulation/detection pairs for IR-UWB systems with and without chirp pulse compression have been investigated. The authors strongly recommend the usage of chirp pulse compression in medical IR-UWB WBAN devices that are expected to use low data rates. Non-coherent detectors for Pulse Position Modulation (PPM) and TR Pulse Amplitude Modulation (TR-PAM) are proposed in [60]. The authors verify via simulations that the knowledge of the average power delay profile at the receiver can substantially improve the system performance.

##### FM-UWB

**Figure 3.**
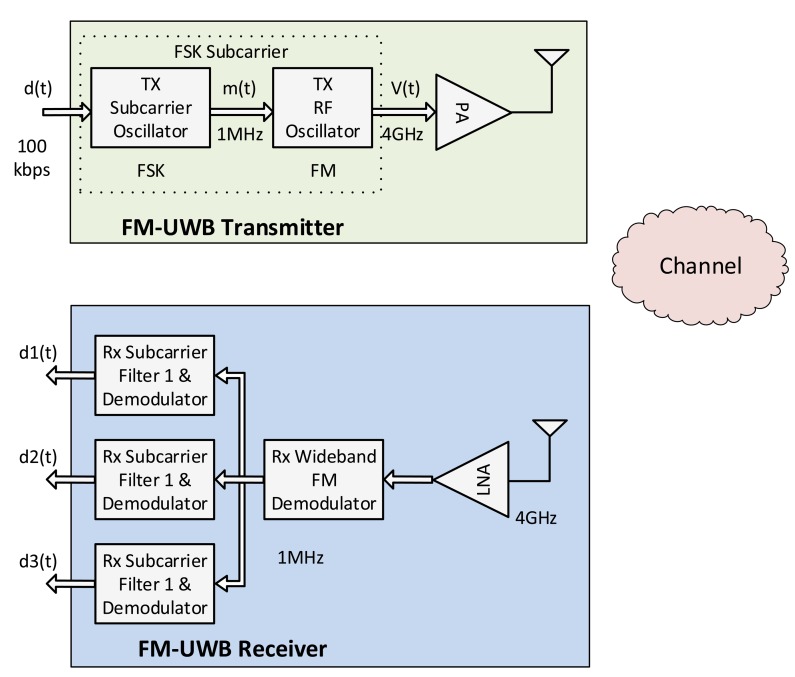
FM-UWB Transceiver.

FM-UWB is a low-complexity, constant envelope, low data rate UWB communication technology with good interference robustness at very low data rates and lower power consumption with respect to IR-UWB. FM-UWB is typically an analog implementation of a spread-spectrum system using double FM: digital Frequency Shift Keying (FSK), followed by high modulation index analog FM to create a constant envelope UWB signal. A typical FM-UWB transceiver architecture is presented in Figure 3. The data signal modulates a low-frequency subcarrier using NB FSK techniques. Then, the subcarrier signal modulates the RF oscillator using analog FM with high modulation index. The receiver demodulates the FM-UWB signal without requiring local oscillator and carrier synchronization (as in the case of IR-UWB), which makes the device both simpler and cost-efficient.

The main characteristics of FM-UWB are: (i) simple and straightforward transmitter implementation; (ii) no need for carrier synchronization at the receiver; and (iii) system operation with a variety of antennas, adding robustness against NB jamming.

A novel system for the monitoring of vital signs based on the FM-UWB technology is presented in [61]. The authors study the error introduced by each block of the transceiver, in order to understand the achievable resolution under non-ideal working conditions. A comparison with an IR-UWB system is also conducted, showing that the FM-UWB system can achieve the same performance, working with lower power levels. The authors in [62] propose the implementation of a device which integrates both sensing and communication capabilities by using the same FM-UWB technology. Finally, the authors in [63] compare the performance of IR-UWB and FM-UWB transceivers in various channel models, showing that while the selection of the channel model has a has a major impact on the FM-UWB performance, the performance of the IR-UWB transceiver is not significantly affected.

##### MB-OFDM UWB

**Figure 4.**
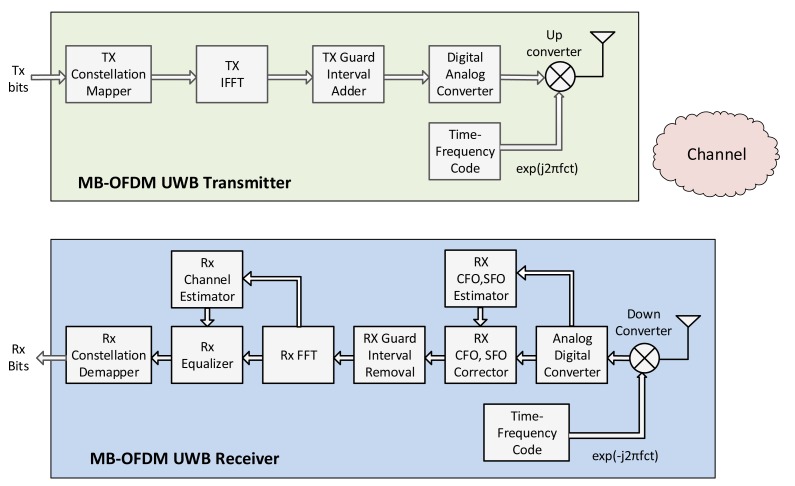
Multi-Band Orthogonal Frequency Division Multiplexing UWB (MB-OFDM UWB) Transceiver.

MB-OFDM UWB is a technique based on the simultaneous transmission of data over multiple carriers spaced apart at precise frequencies. A typical architecture of an MB-OFDM UWB transceiver is illustrated in Figure 4, where the carrier and sampling synchronization is performed after the analog front end. In the specific architecture shown in the figure, time and frequency synchronization (carrier and sampling offset estimation and compensation) are performed in the time domain, while channel estimation and equalization are performed in the frequency domain. Nevertheless, different transceiver designs can be implemented, depending on the adopted algorithms for synchronization, channel estimation and signal detection.

Compared to IR-UWB modulations, MB-OFDM UWB can make a more efficient use of the spectral resources. Moreover, a narrower sub-band bandwidth relaxes the requirement on the sampling rates of analog-to-digital converters, and consequently, facilitates digital processing. The use of OFDM makes the receiver spectrum-efficient and capable of better capturing multipath energy, compared to an equivalent single-carrier Rake receiver using the same total bandwidth. On the other hand, the drawbacks of an MB-OFDM UWB transceiver are that: (i) the transmitter is more complex because it requires an IFFT; (ii) the peak-to-average ratio may be slightly higher than that of the pulse-based multi-band approaches; and (iii) the generation of multi-tones requires high power consumption.

The authors in [64] provide a system-level analysis of MB-OFDM UWB radio systems, by calculating the BER of several on-body radio links measured in an indoor environment. Results demonstrate that the system performance is significantly affected by the position of the on-body sensors, as well as by the changes in body posture, highlighting the importance of considering these parameters when designing optimal MB-OFDM UWB radio systems for WBANs. A thorough investigation of key design issues for MB-OFDM UWB systems, such as FFT size, guard interval size and coding scheme, is provided in [65]. Finally, the NB interference impact on MB-OFDM-UWB systems has been examined both theoretically and by means of simulations in [66], revealing the importance of developing novel and low-complexity NB interference mitigation schemes for OFDM-UWB receivers.

Another big challenge in MB-OFDM UWB is that the constraint of ultra-low transmission power complicates channel estimation. Moreover, the heterogeneous environment of WBANs leads to a dense multipath wireless channel. The authors in [67] present an overview of channel estimation techniques for MB-OFDM UWB communications, highlighting their strengths and weaknesses, and providing useful suggestions for the adoption of these methods for WBAN channels.

#### 4.3.3. HBC Technologies

Unlinke the other two WBAN PHY layer wireless technologies, HBC uses the human body as the communication medium. HBC transceivers enable low-power communication at low frequencies. Previous studies have shown that the optimal operation frequency range for body coupled communications ranges between 10–100 MHz [68,69]. The lower bound is imposed by propagation losses while the upper bound is determined by the fact that the RF energy is no longer confined to the body but is radiated into the environment. Therefore, HBC signals must be modulated to a carrier lower than 100 MHz, before being capacitively coupled to the body via electrodes on or near the surface of the skin. Recently published works on HBC transceivers [70,71] have shown energy efficiency in the order of 1 nJ/bit, data rates of 1 Mbps and receiver sensitivity of −60 to −70 dBm.

The low amount of power radiated outside the body by an HBC transceiver provides a layer of inherent security to the user, allowing spectrum reuse between adjacent users without requiring an explicit cellular structure. A major concern, however, is the interference cancellation due to the presence of high-powered television and radio stations that coexist in the same bandwidth. The authors in [70] adopt a cognitive FSK modulation to avoid frequencies with high interference, whereas the authors in [71] employ a high-input resistance at the receiver to reject offending spectrum above 30 MHz, and a correlation-based scheme to reject signals with low correlation with respect to the expected received signals.

An overview of the presented related work on PHY layer transmission techniques has been provided in Table 3.

### 4.4. MAC Layer Design for WBANs

The main functions of the MAC layer include: (i) the access regulation to the shared wireless medium among the system nodes; and (ii) the scheduling of transmissions to achieve desired performance goals, such as throughput guarantees, time-delay constraints and QoS provisioning. In addition to these functionalities that are common to all systems, a MAC protocol suitable for WBANs must handle specific challenges, associated with the WBAN topology and the node constraints. Furthermore, the MAC protocol design has a significant impact on energy efficiency, QoS provisioning, reliability and interference, which, as mentioned in Section 3, are some key requirements of mHealth applications.

Several extensive surveys and comprehensive guidelines on MAC layer design for the wider category of sensor networks are available in the literature, such as the works in [74,75] and more recently in [76]. Even though these works can provide helpful insights for many of the challenges also faced in WBANs, especially in the design of low-energy MAC protocols, they are not specifically addressed to healthcare applications. Two overviews on WBAN-oriented MAC protocols have been published in 2010 [77,78], but are missing later advancements in this rapidly growing research field. Finally, more recent surveys on WBANs that include discussions on the MAC layer challenges can be found in [15,16,21,22].

In this section, we update and complement the existing surveys by identifying the main challenges and design approaches to tackle the aforementioned requirements of mHealth applications in WBANs. After a thorough study of the proposed MAC protocols in the literature, we provide a survey of the representative mechanisms employed for (i) energy-efficient channel access and synchronization; (ii) QoS provisioning; (iii) reliability; and (iv) interference mitigation and coexistence.

#### 4.4.1. Energy-Efficient Channel Access and Synchronization Mechanisms

In an ideal energy-efficient MAC protocol, nodes would spend most of their time with their radio interface turned off (or in a low-consumption sleep mode) and wake up only to transmit or receive data in a collision-free way and without unnecessary listening times. However, in practice, significant energy is consumed in data collisions and idle listening periods, occurring when the nodes keep listening to the channel, usually after waking up from sleep mode [77]. To reduce power consumption, most WBAN-oriented protocols employ sleep modes. The challenge, however, is to design mechanisms that handle efficiently the periods during which the nodes remain awake. This is mainly achieved through channel access schemes that reduce data collisions and minimize listening times. Synchronization is also an important issue, since most energy-efficient access mechanisms require common time reference within the WBAN. The rest of this section summarizes the main mechanisms adopted in the literature with respect to these issues.

##### Collision-free channel access

Many proposals guarantee collision-free data transmission by introducing low-cycle Time Division Multiple Access (TDMA) scheduling schemes where channel access is managed by a central coordinating node. An example of a straightforward TDMA implementation has been proposed in [79]. The proposed superframe structure starts with the transmission of a beacon by the coordinator, followed by time slots that are allocated to nodes according to a known and predefined schedule that matches their periodic traffic patterns. A more advanced TDMA-based solution for periodic traffic that takes into consideration packet retransmission due to channel errors has been proposed in [80]. In the proposed scheme, nodes are again granted a fixed slot allocation in every superframe, long enough to transmit a data packet and receive an acknowledgment (ACK) by the coordinator. In addition, some extra slots are reserved for packet retransmissions in case of channel errors. In both schemes, nodes wake up at predefined intervals to receive beacons and transmit data.

Another mechanism for collision-free data transmission has been proposed in CICADA (Cascading Information retrieval by Controlling Access with Distributed slot Assignment) [81]. In CICADA, the network is organized in a tree topology, with the coordinator placed at the top. The protocol operation defines two cycles for control and data transmission, further divided into time slots. During the control cycle, a slot assignment scheme is forwarded from parent to child node, starting from the coordinator. During the data cycle, the nodes wake up during their allocated slots to transmit data towards the coordinator, while data is flowing upwards from the nodes at the bottom of the tree hierarchy towards the coordinator.

##### Hybrid channel access

Pure TDMA-based strategies completely eliminate data collisions at the cost of reduced flexibility in scheduling. A more versatile approach is to adopt hybrid schemes that combine different mechanisms for channel access and data transmission. The main concept is to divide time into two phases: (i) a contention-based phase, during which the nodes compete for channel access, in order to inform the coordinator of their bandwidth requirements; and (ii) a collision-free phase, during which nodes can proceed to data transmission, after the resource allocation negotiations that took place in the contention-based phase.

An example of a hybrid access mechanism has been implemented in BodyMAC [82]. The protocol introduces a superframe structure divided into three parts: a synchronization beacon, a downlink part reserved for the transmission of data from the coordinator and an uplink part for data transmitted by the nodes to the coordinator. The uplink is split into a Contention Access Part (CAP), during which the nodes can access the channel through CSMA/CA rules, and a Contention-Free Part (CFP), during which time slots are allocated dynamically to the nodes by the coordinator. In order to gain slot allocation, the nodes must notify their bandwidth requirements to the coordinator during the CAP period. Then, different bandwidth allocation schemes can be implemented for periodic or temporal slot assignments. Sleep mode is supported through a three-step handshake, initiated by the transmission of a sleep request packet by the node during the CAP. The coordinator acknowledges reception with an ACK packet and transmits a sleep mode schedule in the downlink period of the following superframe, containing the necessary sleep parameters.

A different implementation of the same principle is adopted in Distributed Queuing Body Area Network (DQBAN) [83]. The superframe structure in DQBAN is again divided into three parts: (i) the access part, further divided into a number of access minislots; (ii) the scheduling part, further dividing into a number of scheduling minislots; and (iii) the data part, in which collision-free data transmission takes place. Nodes compete for channel access by transmitting small control packets in randomly selected access minislots, and a collision resolution algorithm is employed to resolve any collisions among access requests. After a successful access request, nodes enter a transmission queue and are enabled to transmit their packet in the data part in a collision-free manner.

Finally, an energy-aware hybrid access scheme that exploits energy harvesting at the wireless nodes has been presented in [84,85]. The proposed scheme, HEH-BMAC combines two different access methods: (i) a reserved, collision-free polling access phase for nodes with high energy level and more urgent data; and (ii) a probabilistic random access phase for nodes with low energy level. The key novelty of this scheme is that the duration of the two phases is dynamically determined, taking into account the energy level of the body nodes.

##### Preamble sampling

Another class of protocols adopts a technique often referred to as preamble sampling [76]. The main concept is that the nodes follow independent sleep patterns, spending most of their time asleep, waking up only for short periods of time. While they are awake, they listen to the channel for any beacons transmitted by the coordinator. If a beacon is received, they proceed to transmit their data, otherwise they return to sleep mode. Energy consumption can be reduced by minimizing the idle listening time (*i.e.*, the time spent for waiting to receive a beacon). This can be accomplished by adapting the beacon transmission intervals to the sleep pattern of the nodes.

A mechanism to deal with this issue is based on the negotiation of sleep pattern between the coordinator and the nodes. In [86], for example, a master-slave architecture is assumed and the master keeps track of the sleeping pattern of each slave node. The slave remains in a sleep mode until the predefined sleep time elapses, and then wakes up and waits to be polled by the master. A new sleep time is then defined and the process is repeated. In Traffic-adaptive MAC (TaMAC) [87], the coordinator determines the wake-up patterns of the nodes and stores them in a table. By consulting this table, the coordinator can adjust its polling scheme accordingly and transmit beacons when a node is expected to wake up, thus minimizing idle listening times.

In the previous schemes, the node wakeup times are known to the coordinator. Another approach has been adopted in Traffic-Aware Dynamic MAC (TAD-MAC) [88], in which the coordinator tries to learn the sleeping pattern of the nodes. TAD-MAC operation takes place in two phases. During the evolution phase, the coordinator polls the nodes at different times to see if they have packets to transmit, thus gathering statistics on their traffic load that are stored in a register bank. Different policies are employed for in-body (implantable) and on-body (wearable) nodes. In the first case, a star topology is considered and the register bank is maintained at the coordinator, whereas in the second case, a mesh topology is assumed, and nodes keep statistics for their neighbors traffic. The second operation phase begins after several cycles, when the protocol converges to a steady state and the beacon interval is adapted to the wakeup times of each node.

Finally, another way of coordinating transmissions in order to improve energy efficiency is by employing an additional low-power passive receiver at the nodes. This idea has been adopted in [89], where the nodes are equipped with two radio interfaces: (i) the main transceiver for the transmission of data; and (ii) a passive receiver with ultra-low power consumption, employed as a wakeup radio. During sleep mode, the nodes switch off their main radio interface and maintain only the secondary radio. The nodes exit sleep mode and activate the main transceiver only after receiving a wakeup signal by the coordinator in the secondary channel.

##### Synchronization mechanisms

The implementation of all these energy-efficient schemes usually requires some synchronization between the coordinator and the nodes. A widely adopted synchronization mechanism is the transmission of timestamps within control packets sent by the coordinator (usually beacons or ACKs) [79,80,82,87]. Another approach is proposed in [86], where a predefined time interval, called wakeup fallback time, is employed. In case of communication failure, master and slave reattempt communication after this mutually known fallback time elapses. A more advances synchronization scheme called Adaptive Guard Band Algorithm (AGBA) with Drift Adjustment Factor (DAF) has been implemented within the TDMA-based MedMAC protocol [90]. The AGBA introduces two guard bands in the beginning and the end of each time slot. These guard bands have a variable duration which dynamically increases as time elapses from the previous synchronization point. The DAF is a correction factor employed for the calculation of the guard band duration, taking into account the estimated time offset between the clocks of the nodes and the coordinator. Finally, specific healthcare scenarios can offer unique opportunities for synchronization mechanisms. A typical example is H-MAC [91], a TDMA-based scheme that uses heartbeat rhythm to achieve synchronization. Exploiting the fact that many biosignals share the cardiac rhythm pattern, nodes placed on a single patient can process their sensory data to extract a common time reference, without the need of turning on their radio to receive beacons.

#### 4.4.2. QoS Provisioning Mechanisms

As mentioned in Section 3, healthcare applications have very heterogeneous traffic characteristics. Usually, most applications produce low-rate and periodic traffic, thus encouraging reservation-based scheduling solutions for uplink communication at the MAC layer. However, high traffic applications, such as video monitoring or aggregated data of given sensors (e.g., ECG, EMG, *etc.*) must also be supported. Hence, MAC protocols should define different levels of priority and support advanced scheduling policies, often based on the cross-layer design paradigm.

##### Service differentiation and access priorities

Priority-based scheduling based on service differentiation is one of the most widely employed mechanisms for QoS. The main idea lies in the assignment of different access priorities to traffic flows in order to achieve specific performance goals. Different criteria can be employed for the priority assignment, such as QoS requirements (e.g., latency constraints) or data criticality (e.g., emergency alarms versus normal data traffic).

In the context of WBANs, a common approach is to distinguish between normal and emergency traffic and assign a higher level of access priority to the latter. This concept is adopted in the Urgency-based MAC (U-MAC) [92], a protocol based on the slotted Aloha mechanism employed in the IEEE 802.15.4. U-MAC classifies all traffic flows as either critical or non-critical. Critical data flows are allowed a higher number of transmission attempts, thus increasing their channel access probability. A different way to handle emergency data has been proposed in [86]. The idea is that whenever the master is not busy communicating with a slave that is awake, it sequentially sends polls to all associated slaves. If a slave node wants to communicate an alarm, it stays awake until a poll is received (otherwise, under normal traffic, the slave would wake up according to its sleep pattern). In [87], alarms are handled with the help of a secondary wakeup radio: the node informs the coordinator on the presence of emergency data by sending a control packet in the wakeup radio, and then the coordinator immediately polls the node in the main channel to retrieve the alarm message.

More advanced service differentiation schemes define more priority levels. For example, in BodyQoS [93], three classes of descending priority are defined: reserved downlink data from the coordinator to the nodes, reserved uplink data from the nodes to the coordinator and best-effort data. Reserved downlink data are scheduled first in each superframe, followed by the reserved uplink data, if there is still available bandwidth. Any remaining bandwidth is devoted to best-effort traffic. Adaptive resource scheduling that takes into consideration the channel quality is also provided. Two levels of scheduling are defined in [29]. At the first level, traffic flows are classified according to their criticality level (*i.e.*, high, medium or low urgency). At the second level, each priority class is allocated a dynamically adjusted non-zero portion of the bandwidth according to the Generalized Processor Sharing principle, whereas Earliest Deadline First scheduling is adopted for traffic flows of the same class.

##### Cross-layer scheduling

In addition to service differentiation, advanced scheduling algorithms can be designed by taking into account cross-layer information acquired from the interaction of the MAC with other protocol layers. Cross-layer parameters often include channel state information, provided by the PHY layer, or routing information from the network layer, among others.

A simple channel-aware method, called Flipping algorithm, that can improve the performance of TDMA-based schemes, has been proposed in [94]. The solution applies to channels that can be modeled as a two-state Markov chain: good channel condition ensures successful data transmissions, whereas packet losses occur when the link quality is bad. The Flipping algorithm schedules the transmissions within a TDMA superframe based on the outcome of the previous superframe. All good links of the previous frame are scheduled first, but with reversed order, in order to exploit the most recently observed good quality links. On the other hand, all bad links are scheduled last in the new superframe, thus increasing the probability of having their channel quality changed to the best. BANMAC [95] is a more advanced cross-layer scheme that exploits the fact that, even though the channel quality of WBANs experiences many fluctuations (e.g., due to the movement of body parts), some regular patterns can still be observed. BANMAC is able to predict the channel fluctuations and schedule the transmissions of the nodes opportunistically when the channel conditions are more favorable. Another cross-layer approach is adopted in CICADA [81], where a joint MAC scheduling and routing scheme is employed to ensure ensure low-latency and network flexibility to topology changes.

Although QoS is the main objective of the scheduling mechanisms presented in this section, sometimes scheduling may also result to a reduction of energy consumption. In continuation, we present some schemes that include energy-related parameters such as power constraints (e.g., power limitations of implantable devices) or battery lifetime levels in the scheduling decisions.

In BSN-MAC [96], for example, four priority classes are defined to match four energy levels: (i) unconstrained, for the coordinator node that is assumed to have no energy constraints; (ii) constrained, for wearable sensor devices; (iii) highly constrained, for ingestible sensor devices; and (iv) extremely constrained, for implantable devices. The protocol also defines three levels of data criticality (low, normal and high). These parameters, along with the remaining battery lifetime level and the buffer occupancy, are combined to produce a cross-layer metric used to adjust the length of the IEEE 802.15.4 superframe with the objective of reducing latency while enhancing energy efficiency.

DQBAN takes into account the quality of the wireless link, the residual battery lifetime and the waiting time of the nodes in the transmission queue to implement a cross-layer, fuzzy-logic scheduler [83]. The main idea is that each node can affect the scheduling order by sending a specific request in the scheduling part of the frame. For example, a node may request to be given priority if its energy level becomes critical or to delay its transmission if bad channel quality is detected.

A cross-layer TDMA scheme that aims to increase the battery lifespan of the nodes without compromising reliability and QoS requirements, has been proposed in [97]. The main driver of the proposed scheme is based on battery discharge dynamics which dictate that battery lifetime can be increased if the battery remains idle for a certain amount of time. In order to maximize the idle time between transmissions, nodes may choose to accumulate several packets in their buffers. A decision on whether to transmit in a given time slot is made depending on cross-layer information regarding the battery recovery level, the buffer occupancy and the wireless channel state.

Finally, PEH-QoS [98] promotes energy-aware management and QoS provisioning in a WBAN with energy harvesting capabilities by proposing a three-module architecture that comprises: (i) the Power-EH Aware Management (PHAM), which ensures the optimal use of the harvested energy, by preventing transmission attempts by nodes with insufficient energy levels and encouraging the detection of all events by the sensor nodes (to ensure the detection of critical medical events); (ii) the Data Queue Aware Control (DQAC), which prevents the saturation of the data packet queue and maintains the clinical validity of the data by discarding outdated packets; (iii) the Packet Aggregator/Scheduling System (PASS) that maximizes throughput and energy efficiency by supporting the transmission of aggregated data packets.

#### 4.4.3. Reliability

The reliable data transmission is another key issue addressed in the MAC layer. Since redundancy of devices is not a possibility, given the unique role played by each node in the WBAN, the MAC protocol must provide solutions to increase reliability. This can be achieved by supporting retransmission mechanisms or by increasing diversity through techniques such as cooperative network coding (NC).

##### Retransmission mechanisms

A usual approach is to employ ACK packets to acknowledge the correct data reception (e.g., [82,83,87]). In case of erroneous reception, the data packet can either be discarded or a retransmission can be requested. In [80], for example, extra slots are reserved for packet retransmissions and their optimal number is calculated as a function of the packet error rate.

##### Diversity techniques based on NC

Cooperation between nodes is a key mechanism that can be used to enhance diversity. Exploiting the broadcast wireless nature, nodes in the vicinity of the transmitter (relays) can actively contribute to the communication by forwarding any overheard packets to the destination. Recently, cooperative NC techniques have been studied as a potential way to enhance reliability in WBANs.

The work presented in [99] shows the potential throughput improvement under error-prone channels through the application of network coding, as a function of the number of redundancy packets, the employed relays and the number of sink nodes. More advanced schemes, such as cooperative diversity coding where both coded and uncoded data packets are transmitted, can yield ever better results [100]. A tree topology has been assumed in [101], where multiple nodes communicate with the coordinator through a small number of relays. The nodes send their uncoded data to the relays, which in turn forward a set of both coded and uncoded packets to the destination. The obtained results show that network coding can offer higher reliability with respect to traditional redundancy schemes where multiple retransmissions of uncoded packets take place. Similar conclusions are drawn in [102], where the coding decisions take into account the reliability requirements of different traffic flows.

Finally, a novel cloud-assisted MAC protocol, CLNC-MAC, that employs NC and guarantees the successful delivery of all generated data to the destination through a relay network, regardless of the channel conditions, has been proposed in [30]. The key concept behind CRNC-MAC is to employ cloud resources for the relay coordination, to ensure that all crucial information from the source is propagated without losses through the network (that may include multiple hops), thus enabling the successful decoding at the destination. This is achieved by employing a central coordinating entity, namely the cloud controller, which has the task of verifying the reception of all required information by the relays at a hop by hop basis, requesting retransmissions whenever necessary.

#### 4.4.4. Interference and Coexistence Mechanisms

Interference-aware mechanisms aim to increase the robustness of WBANs to errors due to strong interference sources operating in the same license-free frequency bands, such as IEEE 802.11 or Bluetooth devices, often present in medical environments. Many existing works embrace the concept of cognition, by dynamically selecting the best channel for data transmission from a pool of available frequency bands, based on specific metrics, such as the channel quality or the interference levels. Coexistence with other wireless technologies that operate in the same frequency bands is also a key issue.

##### Interference-aware channel selection

A cognitive radio approach for mHealth services is considered in [103]. Two spectrum sensing mechanisms are proposed to detect channels free of primary user activity. Between sensing periods, beacons are transmitted by the coordinator to distribute periodic bandwidth allocations to the nodes for normal traffic, whereas a small time interval is reserved immediately after the beacon transmission for the communication of emergency events.

In [29], Multi-channel Quality-based MAC (MQ-MAC) is proposed in a scenario where the nodes placed on each patient form a WBAN cluster, and cluster coordinators form a mesh network to forward the aggregated patient data to a monitoring unit. In MQ-MAC, the cluster coordinators create and maintain a table with the link quality of all available channels. For each point-to-point link, the transmitter and the receiver agree on the most suitable channel for their communication and reserve it through a 3-way handshake on the dedicated control channel. This idea is taken one step further in the same work to form the Channel Quality Based Routing (CQBR) scheme, where the route with the best link quality is selected and enable simultaneous transmissions on different channels by neighboring nodes. Co-channel interference provoked by multiple co-located WBANs is also tackled by BANMAC [95], by a fully distributed channel allocation mechanism.

##### Coexistence mechanisms

The Centralized BAN Access Scheme (CBAS), proposed in [104], tackles interference issues in a coexistence scenario with IEEE 802.11 devices. In CBAS the coordinator has cognitive radio capabilities and is able to scan the available spectrum continuously. When a suitable channel is found, the coordinator follows the IEEE 802.11 access rules to reserve the channel for the estimated time required for the data exchange by transmitting a legacy RTS packet. After that, it issues a wakeup command to the next scheduled node on a dedicated secondary control channel, thus waking up the node from its sleep mode and informing it on the data channel selection.

Finally, a different interference-related challenge is explored in [105], where the proposed scheme aims to reduce the Electromagnetic Interference (EMI) produced by WBANs, which can lead to the malfunctioning of EMI-sensitive life-supporting medical devices (such as defibrillators) in a hospital environment. A context-aware solution is proposed, in which a coordinating device dynamically determines the transmission power limits for each communication in the WBAN, after consulting an inventory of the location and the EMI constraints of all electronic medical devices in a given setting. The nodes must content for access by transmitting an RTS in the dedicated control channel. The coordinator replies with a negative CTS if no feasible power scheme is available at that specific time due to either EMI constraints or predicted congestion conditions. Otherwise, a positive CTS with the maximum supported power allocation is transmitted, and the node enters a queuing system, waiting for its turn to transmit in the data channel.

The presented state-of-the-art approaches for the key MAC layer mechanisms, namely energy-efficient channel access, QoS provisioning, reliability, and interference and coexistence are summarized in Table 4.

## 5. End-to-End Solutions for M2M Communication

The goal of an mHealth application is to provide a bridge between the patient and the medical personnel. Hence, the M2M system must provide end-to-end connectivity, connecting the medical sensor devices via the M2M gateway to the Internet, and ultimately to the application server. This role is played by the access communication network that is usually based on WLAN/WMAN wireless technologies such as WiFi (IEEE 802.11), WiMAX (IEEE 802.16) and LTE/LTE-A. Since standardization efforts on M2M communications are still under way, there are many different approaches to achieve end-to-end connectivity.

In this section, we examine end-to-end solutions for M2M communications. In particular, we first discuss theoretical works that focus on the high-level integration of WLAN/WMAN access communication technologies with WBANs. Then, we give some practical examples of end-to-end solutions by presenting testbed implementations for healthcare monitoring. Finally, we present some current research projects for mHealth applications.

### 5.1. Technology Integration for M2M Communications

The integration of access communication technologies with WBANs is a challenging but crucial issue. Some works identify the challenges to enable M2M communications over LTE and LTE-A, focusing on the air interface [106], scheduling mechanisms [107], and the support of low-cost devices [108]. The use of WiMAX-based broadband wireless access technology for mHealth applications, along with potential deployment scenarios and related radio resource management issues has been discussed in [109].

Apart from these high-level approaches, there are some works in the literature that study more specific M2M mHealth scenarios. For instance, [110] discusses the suitability of WiFi-based solutions within healthcare facilities such as single hospital units, as long as proper design, installation and validation methods are followed to meet the specific application requirements.

In [111], a two-tier network architecture is considered. At the lower tier, multiple sensor devices worn by a single patient form a WBAN, by employing the CSMA/CA access mode of IEEE 802.15.4. At the upper tier, multiple WBAN coordinators (corresponding to multiple patients) located within a specific area (e.g., a hospital ward), communicate with an Access Point through WiFi. End-to-end packet delay and access time has been modeled as a function of the number of coexisting WBANs. An extension of this work, in [112], has introduced service differentiation to prioritize high-rate data streams (e.g., EEG data) by providing them contention-free access, whereas CSMA/CA access is given to low-rate streams (e.g., ECG data).

In [113], the authors discuss the feasibility of employing a hybrid network based on WiFi and WiMAX technologies as the access communication network in an M2M system for mHealth services. The integration of the two technologies poses several challenges, mainly related to QoS provisioning, connection admission control, scheduling, and mobility management through seamless vertical handovers. The envisioned heterogeneous deployment scenario considers nodes with either single (WiFi or WiMAX) or dual radio interface and aims to provide wireless connectivity between different subnetworks, including WBANs, home care networks, mobile patients and networks of healthcare providers (such as intranets of hospitals, clinics, drugstores, *etc.*).

**Figure 5.**
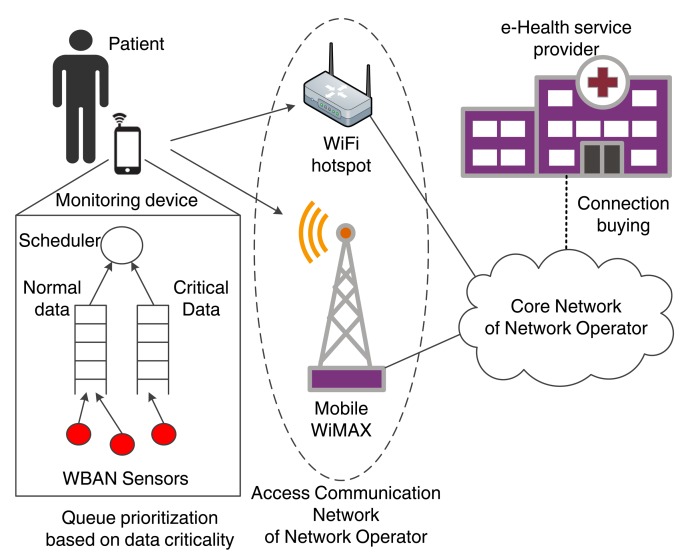
Architecture of remote patient monitoring system for WiFi/WiMAX heterogeneous scenario.

Another remote monitoring scheme that provides ubiquitous connectivity for mobile patients has been presented in [114]. In the proposed scheme, shown in Figure 5, a patient-attached monitoring device collects the WBAN data, classifies them as high-priority (e.g., critical data such as blood pressure, pulse rate and heart rate) or normal priority (e.g., ECG signal) and forwards them towards the healthcare provider through an heterogeneous WiFi/WiMAX access communication network. The access technology is selected depending on the patient's location, considering that WiFi hotspots cover only specific (mainly indoor) locations and WiMAX has a wider (outdoor) coverage. In addition, two types of connections are provided by the network operator: (i) low-cost reserved connections, allocated to patients for given amounts of time (e.g., weeks); and (ii) high-cost on-demand connections, employed when the available bandwidth for reserved connections is not enough to cover the traffic load. The authors approach this mHealth scenario from the service provider's side, who has to buy in advance a certain number of reserved connections from the network operator to serve a given number of patients. Stochastic programming techniques are used to determine the optimal number of reserved connections for each wireless technology in order to minimize the provider's cost.

As far as LTE-based solutions for healthcare applications are concerned, the relevant works in the literature are limited. The impact of 4G communication technologies on mHealth and the emerging challenges are discussed in [115]. In [116], a mapping between the QoS requirements of mHealth services and the existing service classes defined by 3GPP standards is proposed, aiming to provide guidelines for network operators. A cross-layer design for QoS of medical video streaming over mobile WiMAX (IEEE 802.16e) and the 3G High-Speed Packet Access (HSPA) cellular technology is proposed in [117], and a comparison between the performance of the two technologies is given, opening the road to further investigation on LTE-based mHealth applications.

### 5.2. Testbed Implementation of M2M Solutions

Research on end-to-end connectivity is not limited to a theoretical-only level. During the last years, research efforts have been devoted to the actual implementation of M2M networks. In this section, we present the most representative examples of implemented end-to-end solutions for mHealth applications.

In [118], the authors introduce a new file format for the transfer of sensory data and implement a pilot testbed for an end-to-end patient monitoring application. Their contribution is twofold. First, they present an enhanced version of a standard protocol for communication among ECG devices, by proposing an adaptive data structure that can handle multiple vital signals, as well as data for positioning, allergies and demographic information on patients. The definition of a standardized data structure is an important step towards the integration of the medical data measured by the WBAN sensors with various mHealth information systems for monitoring or administrative purposes, belonging to hospitals, individual care-givers, home-care, *etc*. Second, they implement a testbed of an M2M healthcare application for the remote monitoring of patients suffering from heart problems. The patient is equipped with a WBAN formed by a number of wearable sensors, a Global Positioning System (GPS) device and a PDA. The PDA aggregates the sensory and geolocation data, as well as any additional information inserted manually by the patient, and plays the role of the M2M gateway. On the one hand, it employs Bluetooth technology to communicate with the WBAN nodes and, on the other hand, it has mobile ADSL capabilities to forward the data to the remote server located at a hospital facility. To the other end of the system, a portable data acquisition system is considered, consisting of a medical monitor device, a GPS and a laptop with Internet connectivity. Finally, a software application has been developed for the processing and visualization of the data retrieved by the healthcare provider. The pilot testing of the proposed solution on real patients has revealed some very interesting conclusions. From the doctors' perspective, the use of the M2M mHealth system has been an overall positive experience, facilitating the patient monitoring and the collection of data. The patients, on the other hand, have given a more neutral evaluation. Even though they have generally been satisfied by the experience, they have shown more concerns on the wearability of the sensors, the user friendliness of the software application and the data collection process.

A two-tier architecture is considered in [119] to implement a remote monitoring application for patients with Chronic Obstructive Pulmonary Disease (COPD). Bluetooth is used as the WBAN technology for the communication between the sensors and the coordinating node (e.g., a PDA). Apart from the Bluetooth network interface, the coordinating node can support two additional long-range wireless technologies for Internet connectivity with a remote medical server: cellular General Packet Radio Service (GPRS) and WiFi. The authors perform an interesting experiment by measuring system performance metrics of these two upper-tier technologies. The study clearly shows that GPRS and WLAN have complementary power and delay profiles: the energy consumption of GPRS is low but high delays may be observed, whereas WiFi has higher energy cost but lower delays. Based on the observed results, the authors provide guidelines for the design of an adaptive protocol that switches between the two long-range technologies depending on the scenario: (i) WiFi is the recommended technology, when available, especially in emergency situations due to the low data latency. In these cases, the GPRS interface should remain on but at an idle state, employed only to receive incoming calls if needed; (ii) if the WiFi connection is not available, the WiFi network interface should be switched off completely and GPRS should be employed for communication.

A prototype M2M solution built on Android mobile devices is presented in [120]. At the patient's side, wearable sensors are employed to measure physiological data such as ECG and oxygen saturation. The sensor nodes are connected to M2M nodes, which are, in turn, wirelessly connected to the Internet through the M2M gateway. The sensor network employs the IEEE 802.15.4 protocol along with the IPv6 addressing mechanism. At the other end of the M2M system, a server PC collects and processes the sensor data, which are ultimately made available to the medical personnel through a user-friendly Android application. By combining Ipv6 techniques over mobile communication technology, this testbed implementation has provided a practical, scalable and flexible way to make a large amount of biomedical signals accessible online.

**Figure 6.**
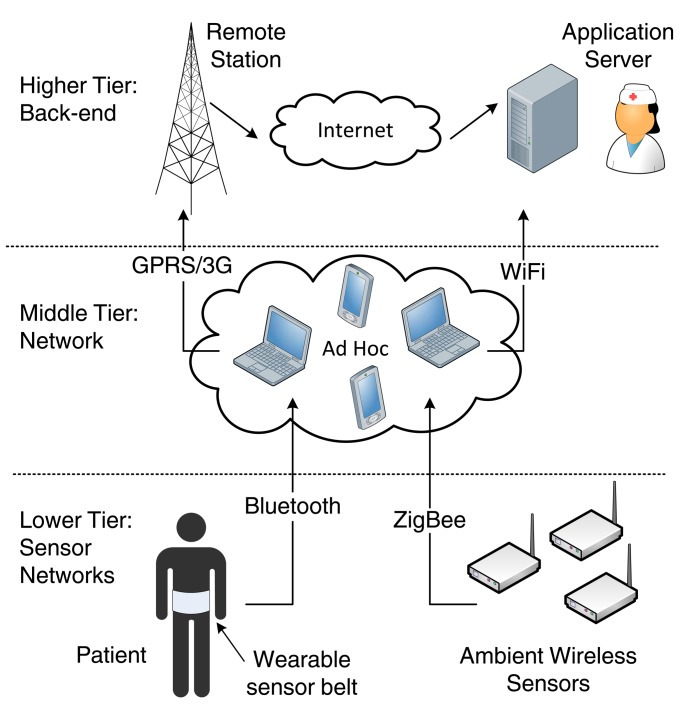
Example of three-tier network architecture.

In some works, ambient sensor networks for environmental monitoring are employed in conjunction with WBANs, in order to provide additional information on the patient's whereabouts, such as temperature, humidity and light conditions. Along this line, a three-tier network architecture, depicted in Figure 6, is proposed in [121], for the remote monitoring of elderly or chronic patients in their residence. The lower tier consists of two systems: (i) a patient-worn fabric belt, which integrates the medical sensors and is equipped with a Bluetooth transceiver; and (ii) the ambient wireless sensors that form a ZigBee network and are deployed in the patient's surroundings (e.g., in the patient's home or in a nursing house). In the middle tier, an ad hoc network of powerful mobile computing devices (e.g., laptops, PDAs, *etc.*) gathers the medical and ambient sensory data and forwards them to the higher tier. The middle-tier devices must have multiple network interfaces: Bluetooth and ZigBee to communicate with the lower tier and WLAN or cellular capabilities for connection with the higher layer. Finally, the higher tier is structured on the Internet and includes the application databases and servers that are accessed by the healthcare providers. The study involves a real implementation of the proposed architecture and tackles several security issues that arise along the three tiers. The proposed framework offers a flexible and secure solution for the monitoring of multiple patients that can be applied to different scenarios, including home, hospital and nursing home environments.

**Figure 7.**
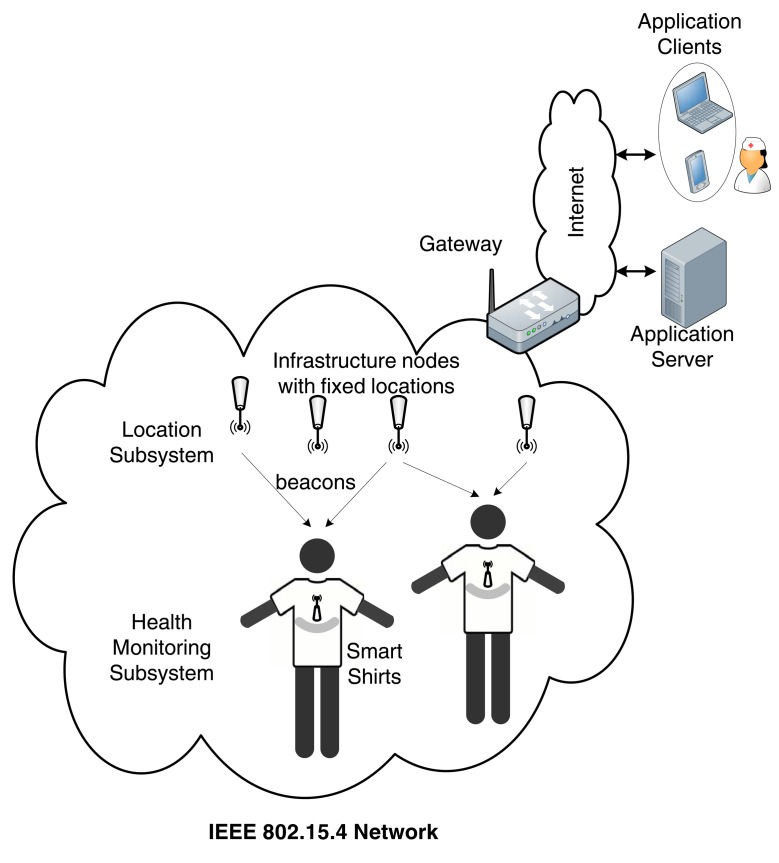
Example of the monitoring and location tracking mHealth system architecture.

Sensor networks can also be employed for patient localization purposes. In [122], the authors propose a system architecture based on two independent subsystems for the monitoring and location tracking of patients within hospital environments, as shown in Figure 7. The healthcare monitoring subsystem consists of smart shirts with integrated medical sensors, each equipped with a wireless IEEE 802.15.4 module. The location subsystem has two components: (i) a deployment of wireless IEEE 802.15.4 nodes that are installed in known locations within the hospital infrastructure and broadcast periodic beacon frames; and (ii) IEEE 802.15.4 end devices, held by the patients, that collect signal strength information from the received beacons. Both subsystems transmit their respective data (*i.e.*, medical sensory data and signal strength information) to a gateway through an IEEE 802.15.4-based ad hoc distribution network. The gateway has wired Internet connectivity and forwards the data to the management server and the monitoring mHealth application. The proposed system has been tested with success in a hospital, achieving high reliability, sufficient battery lifetime of the sensors and real-time data reconstruction. In terms of usability, the obtained feedback of the medical personnel has been taken into account to improve the software interface.

### 5.3. Projects for End-to-End Connectivity

The aim of this section is to present an overview of the most recent and relevant research funded projects for mHealth applications.

**HEALTH@HOME (Health at Home)** [123] aims to provide an end-to-end solution for the remote monitoring of cardiovascular and respiratory patient parameters. The data are continuously gathered through an automatic processing system and are accessed by the responsible medical personnel. A typical client/server architecture is adopted, where the client side is a residential gateway located at the patient's home, able to collect data from the biomedical sensors through wireless Bluetooth links. The most significant measured signals are ECG, SpO2, weight, blood pressure, chest impedance, respiration and body posture. The measured data are sent through the gateway to a server located at the health service facilities that is integrated with the Hospital Information System. The gateway communicates with the server through ADSL as the primary transmission channel, or mobile broadband (*i.e.*, GSM/GPRS/UMTS) as the secondary (backup) data channel. Alarms are sent by Short Message Service (SMS) directly to the physicians, the patients' relatives and their caregivers. The HTTPS protocol addresses the security issues in the communication between the gateway and the server through a certificate validation process. The proposed solution has been tested on 30 patients during monitoring periods of at least one month and has received positive feedback by both patients and medical personnel, as a reliable, user-friendly means of remote control and management of acute conditions.

**HELP: Home-based Empowered Living for Parkinson Disease Patients** [124] targets at designing a health monitoring system able to control disease progression and to mitigate Parkinson Disease (PD) symptoms, thus improving the quality of life of affected elderly people. Although it provides an end to end solution that employs M2M communication for monitoring patients with PD, its aim is to design a control system for a subcutaneous infusion pump that administers the exact required drug dose according to the patients' level of activity without focusing in communication issues. This system is composed of the following components: (i) an intra-oral electronic drug delivery device with miniaturized, non-invasive and removable design; (ii) an external pump that delivers higher amounts of drug; (iii) a WBAN to gather information on the user environment to detect blockades; (iv) a telecommunication and services infrastructure to analyze and transfer data exchanged between the user and the automated system; and (v) a remote care unit for patient supervision.

**CAALYX-MV: Complete Ambient Assisted Living Experiment—Market Validation** [125] goal is to provide an end to end solution that is focused on improving the elder's quality of life. The proposed solution is composed of: (i) a home system capable of monitoring and controlling social and health status of elder people and providing them with some tools and services to support their daily activities; (ii) a roaming system that comprises a smart textile shirt able to measure specific vital signs, detect falls and communicate emergencies; and (iii) a care system for the monitoring of individuals by family, caretakers and health services. All sensors in the WBAN are wearable, measure different parameters such as motion, blood pressure and heart rate, and communicate using Bluetooth links with a mobile phone. The sensory data are sent through standard low-cost networking equipment to a GPS-enabled smart phone (3G/UMTS) that runs a completely autonomous software application. The application continuously analyzes sensor data in order to identify problematic conditions and promptly alert the care system. The proposed system will be validated through 3 pilot programs that will test the usability and acceptability of the system by the users (both patients and caregivers) and will evaluate the reliability and detection accuracy of health problems in the monitored patients.

**Help4Mood** [126] aims at developing an end-to-end system to help the recovery of people with major depression. The system is designed to be used together with other forms of therapy, such as self-help, counseling or medication. The main components include: (i) a personal monitoring system to keep track of important behavior aspects, comprised of sensors for both user activity and sleep monitoring; (ii) an interactive virtual agent asking patients about their health and well-being and providing a portal to trusted health information; and (iii) a decision support system handling the virtual agent to allow its customization to the individual needs of the person with depression.

The sensor devices communicate by using a proprietary low-power RF network protocol named SimpliciTI [127] over Bluetooth. To increase energy efficiency and reliability, the system adopts the idea of cooperation between nodes, achieved through the slight modification of the MAC protocol.

**WSN4QoL: Wireless Sensor Networks for Quality of Life** [128] is a project focused on wireless communication technologies for mHealth applications. The main objectives of WSN4QoL are: (i) to provide a protocol stack architecture, which can accommodate a variety of protocols, algorithms and sensor devices for healthcare applications; (ii) to develop reliable, energy-efficient, interference-robust communication protocols and algorithms; (iii) to develop distributed localization protocols that meet the constraints imposed by WBANs in health care scenarios; and (iv) to propose effective and efficient security solutions for the proposed communication protocols. The proposed protocols and algorithms will be integrated in healthcare commercial devices, in order to evaluate the performance improvements in realistic environments.

To conclude, Table 5 provides a comparison among the main characteristics of the projects presented here. Summarizing, the presented works and projects study different aspects of M2M systems for healthcare delivery, ranging from solving technical communication problems to the implementation of close to market solutions. Despite their differences, several common goals can be identified, that can serve as guidelines for the design of successful end-to-end mHealth applications: (i) miniaturization and enhanced wearability of the sensor devices, to provide unobstructive monitoring that will not interfere with normal life activities of the patients; (ii) reliable two-way communication protocols, that guarantee the prompt and successful delivery of data from the medical sensors to the medical personnel, as well as the reception of medical feedback at the patient; (iii) accurate detection of emergency situations to ensure timely medical intervention in life threatening events. In addition, it is important to maintain a low probability of false positive alarms, to avoid unnecessary hospitalizations and interventions; (iv) advanced security mechanisms to guarantee confidentiality and privacy of the medical data; and (v) user friendly and easy to learn application interfaces, to ensure the successful adoption of the mHealth solutions, given that patients are often elderly people not familiar with the use of technology. Furthermore, the visualization of the monitored data must be done in a clear and helpful way for both the patient and the healthcare providers.

## 6. Conclusions

This paper has provided a systematic study on M2M systems for mHealth applications from a wireless communication perspective. We have, then, focused on end-to-end connectivity in M2M systems. After discussing the integration challenges between diverse communication technologies, we have highlighted different design approaches for end-to-end connectivity through examples of practical testbed implementations for healthcare services. Finally, a list of recent research projects in the context of mHealth has been given, with emphasis on the different technical solutions adopted in each project. Based on the high-level ETSI M2M system architecture, we have first focused on the device domain, by presenting an overview of the recently proposed PHY layer technologies and MAC layer design approaches that tackle the specific challenges of mHealth applications for WBANs.

Concluding, despite the considerable amount of work conducted in the rapidly growing area of M2M communications for mHealth, there are still many open issues to be addressed. Apart from the specific challenges in each aspect of the mHealth systems, efforts must be concentrated on standardization activities, that will enable the market exploitation of the scientific contributions in this field by paving the road for the development of interoperable M2M mHealth solutions.

## Figures and Tables

**Table 1.** Technical characteristics of selected Wireless Body Area Networks (WBAN) sensors for mHealth applications [18,23].

**Sensor**	**Battery Lifetime**	**Data Rate**	**Criticality**	**Application**
Deep brain stimulation	>3 years	1 Mbps	High	Therapeutic benefits for Parkinson's disease, chronic pain, tremor, and dystonia
Hearing aid	>40 h	200 kbps	High	Sound amplification
Accelerometers/Gyroscopes	>1 week	1 Mbps	Low	Measurements on motion detection, acceleration and angular velocity
Pulse oximeter (*SpO*_2_)	>1 week	2 kbps	Low	Measurement of hemoglobin oxygen saturation
Capsule endoscope	>24 h	1 Mbps	High	Imaging of the digestive tract
Temperature	>1 week	2.4 bps	Low	Body or environmental temperature measurements
Electrocardiogram (ECG)	>1 week	9.6 kbps	Medium	Heart waveform characteristics
Electromyograph (EMG)	>1 week	100 kbps	Medium	Muscle movement
Gate/Falls	>1 week	250 kbps	High	Fall detection
Electroencephalo-gram (EEG)	>1 week	100 kbps	High	Brainwave activity
Video/Medical imaging	> 12 h	<10 Mbps	High	Digital video transmission

**Table 2.** IEEE 802.15.6 frequency bands and supported rates.

**PHY Layer**	**Frequency Band (MHz)**	**Bandwidth (MHz)**	**Available Channels**	**Rates (kbps)**
Narrowband (NB)	402–405 (MICS)	0.3	10	75.9–455.4
	420–450	0.32	12	75.9–187.5
	863–870	0.4	14	101.2–607.1
	902–928 (ISM)	0.4	60	101.2–607.1
	950–958	0.4	16	101.2–607.1
	2,360–2,400	1	39	121.4–971.4
	2,400–2,483.5 (ISM)	1	79	121.4–971.4

Ultra-Wideband (UWB)	3,000–5,000	499.2	3	Non-coherent: 394.8–12,636
	6,000–10,000	499.2	8	Differentially coherent: 487–15,600 FM: 202.2

Human Body Communications (HBC)	21 (center frequency)	5.25	1	164.1–1,312.5

MICS: Medical Implant Communications Service; ISM: Industrial, Scientific and Medical; FM: Frequency Modulation.

**Table 3.** Overview of physical (PHY) layer transmission technologies [18,23].

**WBAN PHY Layer Technologies**		**Overview of Proposed Schemes**
**NB Technology**		[52] Novel modulation and demodulation scheme
		[53] Novel hardware design
		[54] Novel modulation and coding scheme

**UWB Technology**	**IR-UWB**	[56] Channel modeling and performance study
		[57] Comparative study of IR-UWB receivers (coherent and non-coherent)
		[58] Channel measurements and channel impact on performance and localization
		[60] Evaluation of novel receiver designs with channel state information knowledge
		[72] Design and evaluation of an UWB transmitter for low-power WBANs

	**FM-UWB**	[61] System design and comparison with IR-UWB
		[62] Integration of sensing and communication devices using FM-UWB to a single device
		[63] Performance study of DS-UWB and FM-UWB under two different channel models

	**MB-OFDM UWB**	[64] Channel modeling and system level evaluation
		[65] Receiver design and investigation of the impact of several design parameters
		[66] Impact of NB interference and a novel mitigation scheme
		[67] Overview of constant envelop and carrier-frequency offset mitigation techniques

**HBC Technology**		[70] Novel modulation and demodulation scheme
		[71] Transceiver design and simulation study
		[73] Baseband system design and simulation study

**Table 4.** Overview of Medium Access Control (MAC) layer mechanisms for Wireless Body Area Networks (WBANs).

**MAC Mechanism**	**Proposed Approach**	**Related Works**
*Energy-efficient channel access*		

Collision-free access	TDMA for periodic allocations	[79,80,90,91,94,97,103,104]
	Tree-based distributed scheduling	[81]

Hybrid access (contention-based/collision-free)	Contention phase for access request	[82]
	Distributed queuing with collision-free data transmission	[83]
	Energy-aware polling and probabilistic hybrid access	[84,85]

Preamble sampling, minimizing idle listening	Polling at predefined wake-up times	[86,87]
	Polling with dynamic learning of wake-up times	[88]
	Polling through secondary receiver	[89]

Synchronization	Timestamp through control packets	[79,80,82,83,87,88]
	Predefined wake-up fallback time	[86]
	Secondary channel	[89]
	Dynamic guard bands and clock drift correction	[90]
	Heartbeat-based time reference	[91]

*QoS Provisioning*		

Access priorities	Two-level priorities (normal, alarms)	[86,87,92]
	Multiple priorities	[29,93]

Cross-layer scheduling	Channel-aware	[94,95]
	Routing-aware	[81]
	Energy-aware	[83,96–98]

*Reliability*		

Retransmission mechanisms	ACK packets and retransmissions	[80,82,83,86,87,89,91,96]

Diversity with network coding (NC)	Transmission of NC packets	[99]
	Cooperative diversity coding	[100,101]
	Cooperative diversity coding with QoS	[102]
	Cloud-assisted NC	[30]

*Interference and coexistence mechanisms*		

Interference-aware channel selection	Cognitive radio approach	[103]
	Channel selection based on link quality	[29,95]

Coexistence mechanisms	Coexistence with IEEE 802.11	[104]
	Coexistence in hospital environments	[105]

**Table 5.** Summary of existing projects on mHealth applications.

**Project**	**Application**	**M2M Area Net.**	**M2M Com. Net.**	**Security**
HEALTH@HOME	Cardiovascular disease	Bluetooth	ADSL	**✓**
HELP	Parkinson disease	Bluetooth	–	**✗**
CAALYX-MV	Independent living	Bluetooth	3G UMTS / WiFi	**✓**
Help4Mood	Depression management	Bluetooth/SimpliciTI	ADSL	**✓**
WSN4QoL	Remote disease management	Cloud-based distribution network	IEEE 802.15.6	**✓**
